# Minding the Cyber-Physical Gap: Model-Based Analysis and Mitigation of Systemic Perception-Induced Failure

**DOI:** 10.3390/s17071644

**Published:** 2017-07-17

**Authors:** Yaniv Mordecai, Dov Dori

**Affiliations:** 1 Researcher, William Davidson Faculty of Industrial Engineering and Management, Technion—Israel Institute of Technology, Haifa 32000, Israel; 2 Senior Systems Architect, Motorola Solutions Israel, Airport City 70099, Israel; 3 Harry Lebensfeld Chair of Industrial Engineering, William Davidson Faculty of Industrial Engineering and Management, Technion—Israel Institute of Technology, Haifa 32000, Israel; dori@ie.technion.ac.il; 4 Visiting Professor, Massachusetts Institute of Technology, Cambridge, MA 02142, USA

**Keywords:** conceptual modeling, cyber-physical systems, cyber-physical gap, object-process methodology, model-based systems engineering, Three Mile Island 2 Accident

## Abstract

The cyber-physical gap (CPG) is the difference between the ‘real’ state of the world and the way the system perceives it. This discrepancy often stems from the limitations of sensing and data collection technologies and capabilities, and is inevitable at some degree in any cyber-physical system (CPS). Ignoring or misrepresenting such limitations during system modeling, specification, design, and analysis can potentially result in systemic misconceptions, disrupted functionality and performance, system failure, severe damage, and potential detrimental impacts on the system and its environment. We propose CPG-Aware Modeling & Engineering (CPGAME), a conceptual model-based approach to capturing, explaining, and mitigating the CPG. CPGAME enhances the systems engineer’s ability to cope with CPGs, mitigate them by design, and prevent erroneous decisions and actions. We demonstrate CPGAME by applying it for modeling and analysis of the 1979 Three Miles Island 2 nuclear accident, and show how its meltdown could be mitigated. We use ISO-19450:2015—Object Process Methodology as our conceptual modeling framework.

## 1. Introduction

Complex cyber-physical systems (CPS) reside and act concurrently in the cyber and physical domains. Such systems mandate awareness, perception, and conception capabilities and technologies. Modern systems and solutions have become ever more cyber-physical, sentient, aware, and intelligent, as they harness the power of affordable sensors, endpoint computing, cloud-based knowledge and artificial intelligence. These facilitate the capability to monitor, control, and engage sensors, actuators and physical agents. Examples include the human body, manned and self-driving cars, household robotics, smart homes, smart facilities and industries, and smart cities.

CPSs consist of a cyber-based segment and a physical segment [1,2]. The cyber segment is designed to manage, monitor, and control the physical segment. The physical segment senses, generates and sends stimuli to the cyber segment, which, in turn, uses them to evaluate the state of the system’s physical segment and, not less importantly, of the environment. Furthermore, the cyber segment is designed to engage the physical segment, to actuate physical components, or to generate some physical impact on the environment.

CPSs still face inherent functional and technical challenges that stem from system-environment perception discrepancy—the system’s or subsystem’s inability to completely monitor, understand, and control its environment or even peer systems. Due to the availability and cohesion of technologies, these challenges are becoming ever more prevalent in commerce, services, and industry, affecting more people more seriously. The cyber segment cannot capture all the aspects related to the state of the environment and the physical segment it controls. It cannot know the state of all the physical elements at any point in space at any given time. The result is a potential mismatch between the perceived or conceived state of the physical segment by the cyber segment and the actual state of the physical world—the environment or the physical segment.

The cyber-physical duality (CPD) is the notion that cyber-physical entities exist dually and concurrently as both physical embodiments and their informatical representations. Cyber-based agents that interact with external entities must therefore maintain an internal model of those entities so they can facilitate, manage, and control the interaction. For the interaction to succeed, the information that the system holds, which is part of its own internal state as an observer [3] about the entity and its state must be reliable and up-to-date.

The cyber-physical gap (CPG) is the difference between the ‘real’ state of the world and the entities within it on the one hand, and the way the systems or subsystems perceive it on the other hand. The CPG disrupts regular system’s dynamics, behavior, functionality, performance, or output because of failure to correctly align the system’s perception of the state of the environment (or the system’s physical segment) with the corresponding actual state. The term CPG has emerged from the long-recognized distinction between the ‘physical world’ and ‘virtual world’ [4,5,6,7].

This discrepancy stems from technological, cognitive, and engineering inhibitors. Machine sensing, monitoring, data collection, transmission, processing, inferencing, and automation all bear various technological limitations. These limitations, in machines as in humans, incur difficulties in maintaining a view of the relevant portion of the world that is sufficiently close to its real state, as the root cause of the CPG problem. Insufficient awareness of this problem by systems and disciplinary engineers and failure to foresee and mitigate these deficiencies is a major contributor to their appearance and adverse effects. This could also result from plain knowledge gaps regarding the problem or solution domain. Substantially, the inability to model, analyze, and design the system for coping with the CPG [8,9] reduces engineering competency and quality during the critical systems engineering phases, such as requirements engineering, system architecting, functional decomposition, risk analysis, technology selection, subsystem specification, and detailed design [10]. Thus, even if knowledge gaps exist, it is difficult to identify and bridge them, and the system remains exposed to the CPG risk.

The combination of technological, cognitive, and engineering limitations has provably led to dire consequences and catastrophes, especially when CPG was involved—even if not exactly described as such [11,12,13]. The current state of affairs has caused and will continue to cause not only regular errors and failures in systems, but also catastrophes such as the 1979 Three Mile Island Nuclear Meltdown [14], and the 2014 Malaysia Airlines MH370 Disappearance [15]. In both cases, there was no knowledge gap—no lack of awareness of the possibility of the event—but the system was not geared for resolving such a problem as it materialized.

There is a need to form an ontology of the CPG and to clarify its importance and ramifications to cyber-physical systems engineers. CPG considerations must be intertwined into normative systems thinking, modeling and engineering. Monitoring, mitigation, and control mechanisms and specification patterns can be incorporated into cyber-physical systems architecture. This will enhance and support CPG risk reduction and mitigation as part of ongoing systems specification and design, reducing CPG threats to the system or the environment.

Scientific, engineering, and operational models have a central role in conceptualizing, explaining, and formulating various phenomena, systems, problems, and solutions [16]. A conceptual model is a knowledge-base: it captures knowledge about the domain. It can also hold information about attribute values or object states of generic types and specific instances. System architects, designers, developers, and operators use models to understand, specify, and communicate the system’s function and architecture (structure-behavior combination) throughout its lifecycle. Modeling importance and significance is further emphasized under high or extreme variability, complexity, and risk [17].

Technological constraints should be well-captured and well-handled as part of system modeling, specification, design, and analysis. Overly simplified or uninformed system models, which neglect or overlook perception factors and impacts of the CPG, result in incompetent systems. An unmitigated CPG can lead to the system’s misconception of its environment, disrupted functionality and performance, risk or failure mode realization, emergency, and ultimately systemic failure, which results in anomalous behavior in the better case, and severe damage or even catastrophe in the worst case. In [18], for instance, a model-based approach for hazard and impact analysis is introduced, but it does not account for potential differences in failure and hazard *perception*, rendering the designed system still exposed to the CPG. The primary contributions of this paper are hence the following:

(i) definition and formulation of the CPG concept and ontology,
(ii) introduction of a formal, model-based, simple, tested, and verified ontological approach for capturing, considering, and controlling the CPG in complex cyber-physical systems, and
(iii) demonstration of CPG in a real-life case with dire consequences, and of the value that CPG-aware modeling and analysis could or would provide in cases of similar nature.

We propose CPG-Aware Modeling and Engineering (CPGAME)—a conceptual modeling approach that provides for capturing, explaining, and mitigating the CPG in complex systems. We argue that such a modeling framework would be most useful and usable as an extension to a common model-based systems engineering (MBSE) framework, rather than as a stand-alone modeling paradigm. CPGAME advocates a holistic systems engineering process, based on a rich conceptual model that covers functional and technical system aspects. This process can describe CPG and potential CPG-induced disruptions to the system and its environment. Accordingly, we employ Object-Process Methodology, OPM [19] as the underlying conceptual-ontological modeling framework for CPGAME. OPM is an ISO-standardized conceptual modeling and simulation methodology—ISO 19450:2015 [20] , a state-of-the-art conceptual modeling and MBSE paradigm [21,22,23], and a proven modeling framework for multidisciplinary, complex, and dynamic systems and processes in science and engineering.

A CPG-aware design may emerge during the modeling and engineering process without an officially-adopted CPG-aware approach. Rather, this may be a result of a lesson learnt from previous projects, a clearly-stated requirement, a compelling necessity, an iterative design insight, or a demonstrated failure in early prototypes. Our purpose in this paper is to make the evolutionary conceptual modeling, design, and engineering process more conscious, effective, expressive, and informative by accounting for the CPG problem and modeling elements to solve it.

The rest of this paper is organized as follows: Section 2 includes a literature review regarding the CPG and various synonymous and analogous concepts, and the state-of-the-art in CPG modeling and analysis. It also includes a brief introduction of OPM, our underlying modeling paradigm. Section 3 includes a specification of the CPGAME approach, including an enhanced modeling process and a modeling pattern that covers the various aspects related to the CPG and its mitigation. In Section 4, we demonstrate CPGAME on the Three Mile Island nuclear reactor partial meltdown accident of 1979, known as “TMI2”, which constitutes a benchmark case in system safety and risk analysis, and a compelling example of a CPG-induced catastrophe. We discuss the results, conclude this paper, and propose directions for future research in Section 5.

## 2. Related Work

### 2.1. Observation and Control

In Control Theory, the difference between a controlled entity’s state and the controller’s state-perception thereof is inherent in the control problem, due to noise, latency, uncertainty, etc. The controller continuously corrects the commands to the controlled entity based on output observations (e.g., using sensors), state inferring based on the outputs, and future-state prediction [24]. Observability is the notion that an entity’s state can be inferred from its output, while controllability is defined as the ability to transfer the entity’s state from any initial assignment to any final assignment within a finite (and feasible) timeframe [24,25]. While observability is critical for determining the state of the controlled entity, controllability is critical for actuating or interacting with that entity.

While observability and controllability are necessary conditions for the solution to almost any control problem, they are usually not sufficient for complex systems, and naïve theoretical reliance on these attributes could result in failures and safety issues [26]. In addition, observability is not always applicable, due to the inability to access all the output or state variables of the controlled entity, or due to invalid inference of states from observed outputs. In [27], deficiencies were pointed-out in brain activity monitoring and inference, due to erroneous assumptions on the bidirectional mapping of cognitive processes to brain activity, or on brain activity being random rather than fractal. In [28], a method was proposed for determining the critical state-variables, indicative output-variables, and minimal output observation sensors for an arbitrary nonlinear dynamical system. In [29], a method was proposed for ensuring the observability of a physiological system—such as the human body or human brain—by determining the minimal number of measurements and sensors for discrete-time fractal statistical analysis. Modern ubiquitous computing ecosystems and the Internet of Things (IoT) paradigm pose new challenges for observability and controllability of dynamic and evolving entities, such as mobile devices, connected things, and intelligent agents [30].

### 2.2. Cyber-Physical Gap: The Discrepancy between Reality and Its Conception

CPG has been a primary concern in cybernetics, informatics, epistemic logic and knowledge representation for a long-time even if not named this way before. Its role in natural, societal, and technical processes is acute [4,6,31,32,33]. The detection and representation of real-world events and information has been addressed in and applied in domain such as system safety [11,12,34], cyber security [35,36,37], and counter-terrorism [38,39]. Recent advances in intelligent physical systems (IPS) have also raised the need for models for self- and environment-perception, observation, and activation, especially in the domains of microbiology and biochemistry [40]. The precise term *Cyber-Physical Gap* has recently been mentioned with respect to the Internet of Things [41], as web browsers’ inability to interact with device-integrated sensors and actuators, and the resulting limited context-awareness. Nevertheless, we found this usage of the term quite narrowing, relative to the potential scope it could apply to.

CPG results at least in part from the limited or missing ability of system models to precisely capture the intricacies of the systems they specify. Different models present different views related to various aspects of the system, such as CPG. Model quality and fidelity is not guaranteed, and models often provide poor information and facilitate little understanding of the systems they describe. This system-model discrepancy is the basis for possible approximation-complexity tradeoff and resulting multiplicity of and variability among several models of the same problem [42,43]. The hierarchical detail decomposition approach to complexity management is the strategy OPM employs to resolve the clarity-completeness tradeoff—the need to provide a clearly understandable system specification on one hand and a complete one on the other hand [19].

The literature on CPG-related aspects does not provide a holistic model-based approach to incorporate CPG notions such as detection, classification, representation, prevention, and mitigation into the conceptual model, architecture, design, and specification of the CPS. As long as this problem is treated without reference to or integration with the core system model, the ability to mitigate the CPG by design would be severely limited. Worse, considering CPGs as features of a *black box CPS*, i.e., without delving deep into the CPS’s internal parts in order to understand, address, and resolve the root causes and impacts, transfers the responsibility to contain and mitigate adverse CPG-related impacts by-design from the CPS to the systems that interact with it and human users or operators.

Our study of CPG and CPG-aware systems modeling, analysis, and engineering included theoretical and fundamental principles of the physical-informatical essence duality (PIED) of cyber-physical entities, and PIED-aware conceptual modeling semantics [8] and integration of CPG considerations into automated decision-making [44] and systems-of-systems interoperability [45]. We also analyzed the occurrence of CPG in various cases, including the lost baggage problem [9] and cyber threat detection and response [46]. The CPG in air traffic control was also proposed as a major factor in the disappearance of Malaysia Airlines flight MH370 in the Indian Ocean in 2014 [9]. The need to provide a holistic modeling framework for defining and managing CPG, as part of a broader disruption-informed modeling and analysis framework [47], has motivated the additional research that resulted in the present paper.

### 2.3. Object-Process Methodology (OPM)

Model-Based Systems Engineering (MBSE) is the execution of engineering activities throughout the lifecycle of complex systems while closely relying on conceptual modeling to describe and specify the evolving system. OPM is an official ISO standard (ISO 19450) [20], one of several leading, state-of-the-art methodologies for MBSE, and a notable modeling language next to languages like the Unified Modeling Language (UML), UML’s systems-oriented profile—SysML, Enhanced Functional Flow Block Diagrams (EFFBD), Mathwork’s Simulink, State-charts, Integrated Definition Framework (IDEF), and others [22,23,48].

OPM features a unique capability to capture functional, structural, and procedural aspects of any natural or artificial system in a unified manner. OPM is founded on the universal ontology principle, whereby the universal ‘vocabulary’ includes the concepts ‘object’, ‘state’, ‘process’, and ‘relation’. Accordingly, this concept set constitutes a necessary and sufficient set of elements for conceptually specifying systems and phenomena in any domain in the universe. Attributes, for instance, are objects that are exhibited by other objects or by processes. Conditions, events, and triggers are enabling relations between object states and processes, eliminating the need for distinct model elements for these concepts.

OPM is bimodal—OPM models include synchronized graphical and textual modalities. OPM’s compact set of graphical-textual elements includes **Things** and **Links (Monospace** font represents names of OPM things): Processes, **Objects** (bold), and *states* (italic) of **Objects**. Processes are represented by ellipses in the OPD. **Objects** are rectangles. *States* are rountangles within **Object** *rectangles*. **Things** inherently exhibit **Essence**, which can be *physical* or *informatical*. In addition, **Things** have **Affiliation**, which can be *systemic* or *environmental*. **Links** express various relations between **Things**. **StructuralLinks** support the modeling of static system aspects. **ProceduralLinks** express procedural relations, control, and causalities.

OPM’s graphical modality is a hierarchical structure of interconnected Object-Process Diagrams (OPDs). The gradual exposure and elaboration of details from one OPD to its predecessors enables complexity management and complicatedness reduction. OPDs are derived from higher-level OPDs by several approaches: (i) unfolding objects and processes for structural or functional decomposition; (ii) zooming into objects and processes for spatial or temporal ordering; and (iii) generating dedicate views for pivotal things. In addition, object states can be suppressed or expressed as needed for clarity and simplification. These mechanisms apply at any depth level, making this elaboration process repetitive and methodical, and making all the OPDs at any OPM model level self-similar. Any construct that appears at least once in some OPD in the model, is valid throughout the model.

OPM’s textual modality is a structured textual specification in Object-Process Language (OPL), an English-like language. Each OPD is accompanied by an OPL paragraph. Each graphical OPD construct is expressed by a semantically equivalent textual OPL sentence.

OPCAT is a freely available OPM CASE desktop modeling and simulation software application (which can be downloaded from http://esml.iem.technion.ac.il/) [49]. OPCAT supports most of the OPM ISO-approved notation. It automatically validates model edits according to OPM grammar, and automatically generates OPL text for each OPD, for each graphical construct. OPCAT provides model execution and simulation capabilities, which support model validation and verification [50].

OPM’s simplicity on the one hand and its expressive power on the other hand enable clear and concise modeling and architecting of complex systems and interactions. This combination, in conjunction with the automated text generation, concept and behavior simulation, concurrency, and physical-informatical essence distinction, are significant justifications for adoption OPM as a modeling and simulation vehicle for the CPGAME pattern and for CPG-aware systems in general.

## 3. CPG-Aware Modeling & Engineering—CPGAME

### 3.1. CPG Awareness

A system’s knowledge base (KB) includes what the system’s cyber-based software or human agent has to know about the physical environment and the types and classifications of entities therein. It is mostly a static collection of general classifications and abstractions. A system’s information base (IB) includes what the system dynamically knows during its real-time operation about the specifics of the physical environment and entities therein, based on information it has gathered or received [47]. The KB and IB are both stored and managed in a database (DB), which may often lead to confusion between them. A CPG-aware approach emphasizes the knowledge-information distinction. Knowledge is what the system conceives as the possible relevant world. Information is what the system perceives as the relevant known world in real-time or a while later.

Based on the premise that stateful objects and processes are necessary and sufficient to represent systems and on the KB-IB distinction, we argue that for the existence and state of *specific* objects and processes to be in the IB, i.e., in the system’s *perception*, the possibility of existence of relevant *kinds* of objects, states, and processes must first be in the KB, i.e., in the system’s *awareness*. Table 1 defines a mapping of KB-level awareness and IB-level perception by kind, feature, and state-space concepts. Awareness refers to knowing about the possibility of a kind, a feature, and a state-space to exist, while perception refers to the system being informed about a specific kind, feature instance, or state. Feature instance refers to non-unique features. For instance, if a system is aware of the possibility of a car to have doors, then perception refers to the number of doors and identity of each door.

With respect to the awareness-perception distinction, we observe three classes of CPG, which apply at the various conception levels, and represent three phases of CPG-related failure. These three classes of CPG, as well as the two types of each class that represent the causes for the failure, are summarized in Table 2. While Table 1 defines the conception of the entity, the outcome of the system’s interaction with the entity is a separate entity of its own. Therefore, while Class A and Class B cover the existence and state of the entity, respectively, Class C applies to the interaction’s outcome.

Humans and intelligent agents might be able to detect and infer the state of external entities based on intrinsic and exogenous sources of information. The internal representation of the external entity by the system may therefore be fuzzy, consisting of several possibilities, each with its probability or likelihood [51]. When several sources of information are available, they can yield inconsistent or even contradicting results. For example, two sensors in a carwash tunnel can indicate different trunk configurations for arriving vehicles: one sensor can sense “hatchback” while the other—“sedan”. The system must therefore include a dedicated mechanism for fusion, reconciliation, conflict-resolution, selection, or determination over the possible state-set of the uncertain entity or one of its properties. One solution can be voting among three sensors. Alternatively, in some cases, a fuzzy representation of the entity may be applicable. Fuzzy existence means that there is a probability *p* for existence and probability 1 − *p* for inexistence. Similarly, the entity’s state may become a probability distribution over a state-set [52]. The performance of the decision mechanism that combines several or multiple information sources can improve over time if machine learning is applied [53]. Conflict resolution may require human intervention in the loop to help an automated agent make its final decision or to enforce a judicious decision on the system. In some cases, the inference process might be too complicated for a non-human agent to resolve.

### 3.2. CPG-Aware Modeling with OPM

The transition from a nominal, or naïve model, towards a CPG-aware model is a gradual process of evolutionary conceptual modeling. During this process, the system architect must identify cases of CPG in the system-environment interaction and incorporate into the model the notion of CPG along with the system’s response to it. In this section, we gradually apply the principles and guidelines of CPGAME in order to evolve the model from naïve to CPG-aware. We use OPM as our conceptual and ontological modeling framework, and define a generic pattern model with the following features:

(a) It expresses the CPD and the various CPG forms on top of the nominal system model.
(b) It specifies the monitoring, control, and mitigation mechanisms to be incorporated into the CPS in order to cope with and bridge the CPG.
(c) It represents the various precarious situations caused by the CPG and how they can be resolved by executing the model via animated simulation.
(d) It is cyclical, time-dependent, stateful, and event-driven i.e., each iteration accounts for the passing of time, the last system state, and the occurrence of new external events.

**Figure 1:**
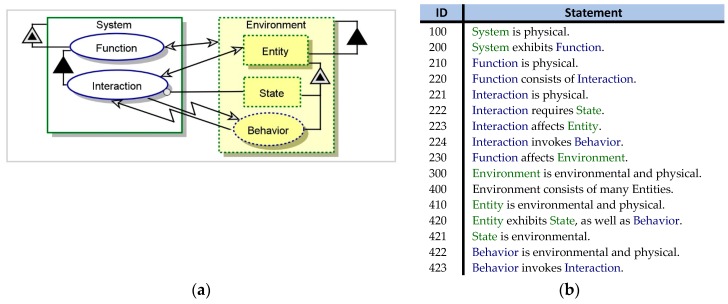
System diagram of a generic naïve system-environment interaction: OPD (**a**) and its equivalent OPL text (**b**).

We begin with a generic system-environment interaction model (M_0_). Figure 1 shows the *system diagram* (SD)—the top-level OPD—of M_0_ in graphical and textual modalities. M_0_ shows an Interaction of the **System** with an **Entity** in the **Environment**, as part of the Function of the **System**. The Interaction is based on the actual **State** of the **Entity**. It invokes Entity Behavior, and is cyclically invoked by it.

M_0_ is defined as a naïve model. It contains an implicit assumption that the system can interact with the environment based on the environment’s actual state. In reality, however, the interaction is based on the internal representation of the environment as sensed and interpreted by the system, giving rise to a potential CPG. To account for CPG, the model has to include an explicit definition of that internal representation of the environment and consider cases of misrecognizing elements in it.

**Figure 2:**
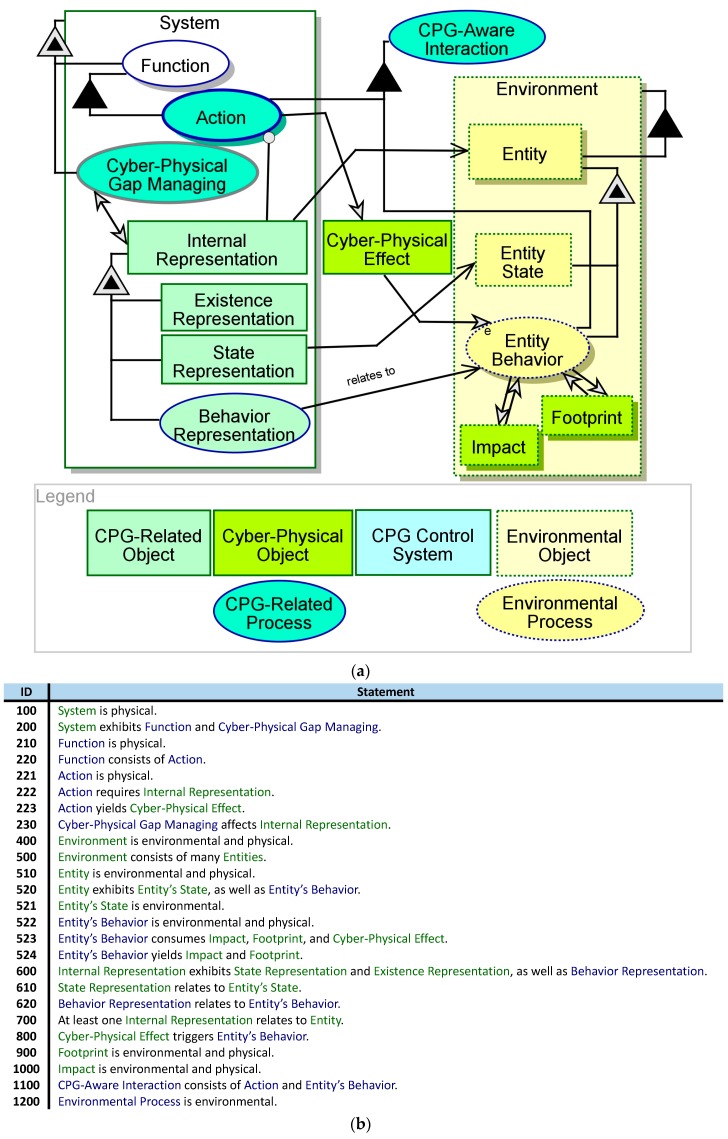
System Diagram (SD) (**a**) and its equivalent OPL text (**b**) of a CPG-aware system.

Figure 2 presents a revised SD, in which the CPG aspect is weaved into the model. The generic CPGAME model is available online at https://1drv.ms/f/s!AsN2SH2tvCOWjmu6bRvpHS1pyiWm (usage is permitted under the terms of the CreativeCommons CC BY-NC 4 license). This enhanced model illustrates the interaction between the **System** and its **Environment** through the **CPG-Aware Interaction** process, which emerges from both the Action of the **System** and the environmental Entity Behavior. The Action generates a **Cyber-Physical Effect**, which triggers Entity Behavior. The **Entity** generates a *physical* **Footprint** of its presence, and a *physical* Impact on the **Environment**. The system must detect and understand both the **Footprint** of and the **Impact**. The Action of the **System** relies on the **Internal Representation**, which relates to the external **Entity**. This **Internal Representation** includes **Existence Representation**, **State Representation**, and Behavior Representation. The functionality for handling these representations is contained in the conceptual process Cyber-Physical Gap Managing.

The CPG-aware model demonstrates a new second-order modeling pattern: the **EnvironmentRepresentation** that the **System** holds is an *informatical* **object** inside the model that is also the model used by the system in run-time. OPM’s metamodeling capability significantly helps this modeling approach; OPM provides the systems engineer with the means to model both the system as a whole and the internal model of the environment that the system maintains. The modeling language for both modeling layers is one and the same.

**Figure 3:**
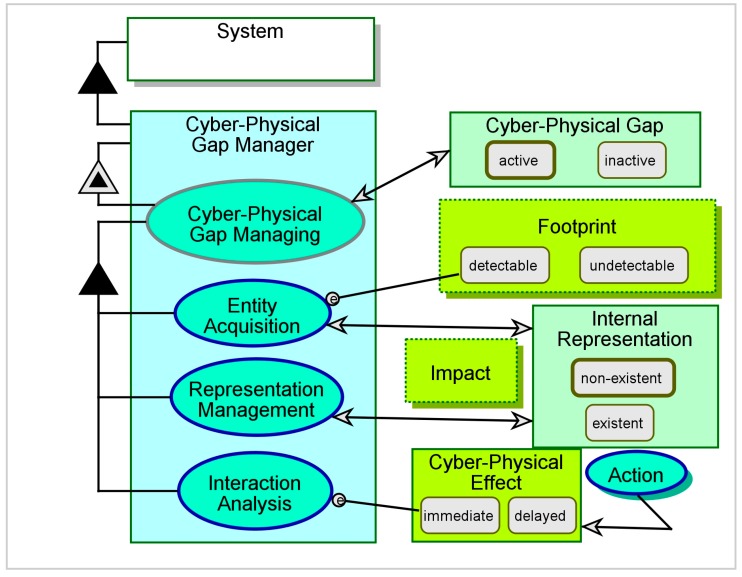
The Cyber-Physical Gap Managing process unfolded.

In Figure 3, Cyber-Physical Gap Managing from Figure 2 is unfolded to reveal three subprocesses: Entity Acquisition, Representation Management, and Interaction Analysis. There is a clear reliance of these processes on the system-maintained **Internal Representation**, in addition to the entity’s **Footprint** and **Impact**, in order to capture the external **Entity** or interact with it.

A CPG can emerge at any phase during the system’s operation. The system may fail to acquire an external entity even if the entity is present in the scene, and consequently disregard that entity. The system can misidentify or misrepresent an acquired entity, and therefore mishandle it. Finally, the actual outcome of an interaction with the entity may not match the outcome that the system expects. A CPG-aware system must implement acquisition, representation, and interaction to cope with these challenges and be resilient to the CPG’s undesired results. In what follows, we specify the CPG-aware approach to these three critical functionalities.

### 3.3. Entity Acquisition

Entity acquisition is the system functionality for detecting entities in the environment. Acquisition must occur before the system can interact with the entity. Assuming system awareness of any such entities by default is wrong and misleading. This assumption is hardly during early design stages for static relations, such as fixed interfaces among fixed and reliably connected subsystems, and more so for complex ones. For a system to become resilient to subsystem connectivity issues, these considerations must eventually be incorporated into the design. As the design progresses, it will be wiser to assume that subsystems may be unaware of the existence and state of each other, let alone external entities.

The issue of awareness and acquisition of external subsystems is also challenging in the context of agent-based systems. An agent in this context is any loosely-connected system or device that has to connect to a central control system in order to commence and maintain some valuable interaction. Consider, for instance, a mobile device with a navigation application that connects to a central application server in order to obtain map and traffic updates, report its position and speed, send route requests, and receive route suggestions. For these interactions to take place, the central control system and each individual device first must become aware of its peers. Controlled, off-line binding may be useful when a manageable number of agents are deployed, but in general one must not assume that two endpoints are aware of each other by default and can interact without a prior check.

The third case for entity acquisition concerns transient entities with which the system may interact. Here are three examples: (a) Baggage items pass through an airport baggage handling system, which must determine their destination according to the barcode that is taped to them; (b) Airplanes travel through an air traffic control (ATC) area, and the ATC system must identify, track, and guide them; (c) Customers walk into a shopping mall or bank branch and are interested in special offers, sales, or services. In all these cases, the system must include suitable means to identify and acquire transient entities with which it is required to interact.

**Figure 4:**
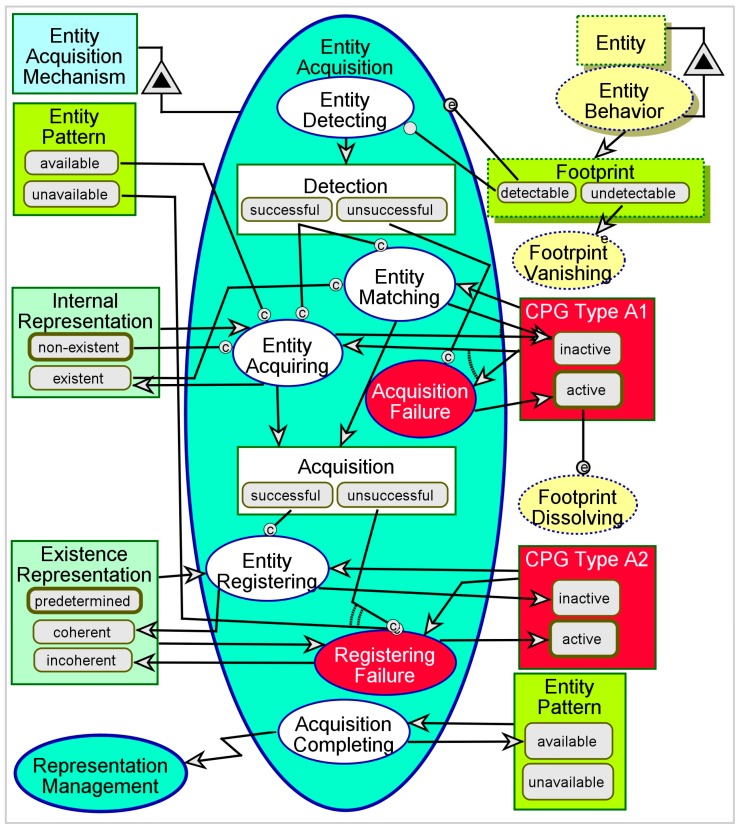
The Entity Acquisition process.

Figure 4 is a model of a generic Entity Acquisition. The process begins when a *detectable* **Footprint** is generated due to the external, uncontrolled Entity Behavior. Even if the **Footprint** is *detectable*, the Entity Detecting phase (the first subprocess) may result in either *successful* or *unsuccessful* **Detection**. An *unsuccessful* **Detection** generates **CPG Type A1**—failure to detect a detectable impact, due to failure in the detection mechanism. A *successful* **Detection** leads to Entity Matching, if an **Internal Representation** is already *existent*. Otherwise, Entity Acquiring generates an *existent* **Internal Representation** upon first detection of the entity. This can only be done if the **Entity Pattern** is *available*—i.e., if the **Footprint** of the external **Entity** is sufficiently similar to an existing pattern. Like **Detection**, **Acquisition** can also be either *successful* or *unsuccessful*. When **Acquisition** is *successful*, it leads to Entity Registering, which generates a *coherent* **Existence Representation**—at this point this is merely acknowledging the existence of an external entity whose **Footprint** matches some existing pattern. When **Acquisition** is *unsuccessful*, this results in **CPG Type A2**—failure to acquire a detected entity. Acquisition Completing invokes Representation Management, in which the details of the representation are treated. A learning system also updates the **Entity Pattern**, so that even if **CPG Type A1** is *active*, it might be neutralized the next time a *detectable* **Footprint** is successfully detected by the system.

To simulate Entity Acquisition using OPCAT in simulation mode, we begin with activating Entity Behavior. This will randomly generate either *detectable* of *undetectable* **Footprint**. An *undetectable* **Footprint** will trigger Footprint Vanishing, which will then consume **Footprint**. A *detectable* **Footprint** will initiate Entity Acquisition. Entity Detecting will randomly result in either *successful* or *unsuccessful* **Detection**. In the first time Entity Acquiring will occur, provided that: (i) **Detection** is *successful*; (ii) **Entity Pattern** is *available*, and (iii) **Internal Representation** is *non-existent*. **If Internal Representation** is already *existent*, Entity Matching will occur instead. If **Detection** is *unsuccessful*, Acquisition Failure will occur, and the Entity Acquisition process will terminate. Entity Acquiring and Entity Matching both result in either *successful* or *unsuccessful* **Acquisition**. If **Acquisition** is *successful*, Entity Registering will occur. Otherwise, Registering Failure will occur. After each completion of Entity Acquisition, the process can be repeatedly simulated by re-activating Entity Behavior. Various combinations of output variables will be generated, including four possible combinations of **CPG Type A1** and **CPG Type A2**.

### 3.4. Representation Management

Representation management allows the system to develop a sufficiently precise understanding of the acquired entity’s state and behavior, and refine the internal representation so it can support system-entity interaction. The representation of the entity evolves and improves with each cycle of analysis of the entity’s behavior, its footprint, and its observable attributes. Wrong interpretation of these properties of the entity may mislead the system. Fusion of multiple sensors or data sources, along with machine learning and proactive monitoring and data collection should be applied, in order to (a) reduce the likelihood of inference error; and (b) converge towards reliable and precise representations.

**Figure 5:**
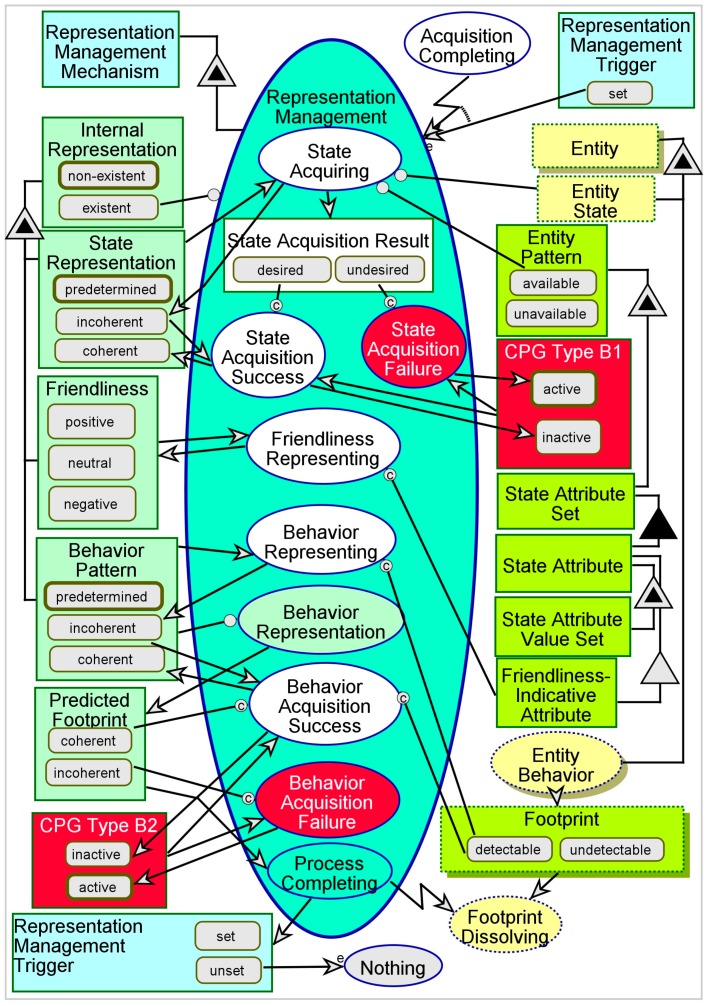
The Representation Management process.

A model of the Representation Management process is shown in Figure 5. The process begins with an invocation by the previous process—Entity Acquisition—or by self-reactivation of the process by itself, using the **Representation Management Trigger**. First, State Acquiring occurs, and the values of state attributes of the entity are acquired according to the **State Attribute Set** stored as part of the **Entity Pattern**. Not all the attributes of the entity may be acquired, and those state attributes, which are critical or necessary for successful interaction, must be defined in the system’s KB so that it may track them. It is necessary that all the **State Attributes** be acquired correctly for the **State Acquisition Result** to be a *desired* one, which qualifies the **State Representation** as *coherent*. Otherwise, it is an *undesired* result, and the **State Representation** remains *incoherent* as initially assumed. This reasoning is projected as the **CPG Type B1**—failure to acquire the state of an acquired entity.

Transient entities may be friendly, passive, or adversary. A friendly entity, such as a mobile device or remotely operated semi-autonomous vehicle, may communicate with the central control and provide details on its whereabouts and intentions. A passive entity, such as a barcode- or RFID-tagged baggage, cargo, or merchandise, may only react to attempts to communicate with it and respond as needed. An adversary entity, such as an intruder, malicious software, hostile aircraft, or attacking ballistic missile, may try to evade the system, refuse to communicate, mislead and confuse it, and even disrupt or attack the system directly. In many cases, the system must include suitable means to determine the friendliness classification for the transient entities that it acquires. This is especially critical in environments that host a mixture of friendly and adversary entities, such as a battlefield, air traffic corridors, or computer networks.

The Friendliness Representing process determines whether the acquired entity’s **Friendliness** is *positive*, *neutral*, or *negative*. This optional process must be implemented in cases like the abovementioned ones. The system must hold some **Friendliness-Indicative Attribute Set** as part of the **State Attribute Set** to determine **Friendliness**. Designers must be aware of the possibility that such attributes may be exploited by an adversary to mislead the system to consider an entity friendly or neutral. Hence, friendliness evaluation may also be susceptible to a special case of **CPG Type B1**.

The third part of Representation Management includes the representation of the entity’s behavior. The Entity Behavior is inferred from its *detectable* **Footprint**—the same **Footprint** that triggered the Entity Acquisition process. The system derives a **Behavior Pattern**, which is injected into the Behavior Representation model that the system holds as part of the **Internal Representation**. Behavior Representation is executed by the system and a **Predicted Footprint** is generated. This is where models for near-future state prediction, such as Kalman Filtering [54] can be applied. If the **Predicted Footprint** is *coherent*, i.e., consistent with the **Entity**’s *detectable* **Footprint**, this means that the inferred **Behavior Pattern** is also *coherent*. Otherwise, this means that the **Behavior Pattern** is *incoherent*—a situation defined as **CPG Type B2**. When Representation Management Process Completing occurs, it determines whether another Representation Management iteration is needed to refine the results, and generates the **Representation Management Trigger**, which triggers another iteration if it is *set*. Considerations for additional iterations are however beyond the scope of the current paper.

Simulating Representation Management using OPCAT must follow at least one successful completion of Entity Acquisition, so that the **Internal Representation** will be created. As explained, activating Entity Behavior and generating a *detectable* **Footprint** will allow State Acquiring, Friendliness Representing, and Behavior Representing. The model is designed to generate random results for internal control/decision variables such as **State Acquisition Result**, **Predicted Footprint**, and **Representation Management Trigger**. Consequently, any of the four possible combinations of **CPG Type B1** and **CPG Type B2** may emerge.

### 3.5. Action and Interaction

Cyber-physical systems function to obtain their goal or serve their purpose by interacting with entities within the environment. Therefore, we first specify the actions and interactions from a naïve, value-providing vantage point. Nevertheless, applying the CPGAME approach means that any interaction is based on an internal representation rather than on an external state that is assumed to be known to the system. A mismatch between the internal representation and the actual manifestation—a CPG—may result in incoherent system behavior or entity’s response. The model must therefore specify the disrupted, CPG-aware case next to the nominal one, in order to clarify the ramifications of incoherent action and interaction, providing the following benefits:

(a) Understanding of the implications of incoherent actions,
(b) Ability to simulate, map, and analyze the possible paths, especially those leading to failure,
(c) Compliance with regulations or safety requirements, and
(d) Incorporation of engineering or operational risk mitigation mechanisms.

**Figure 6:**
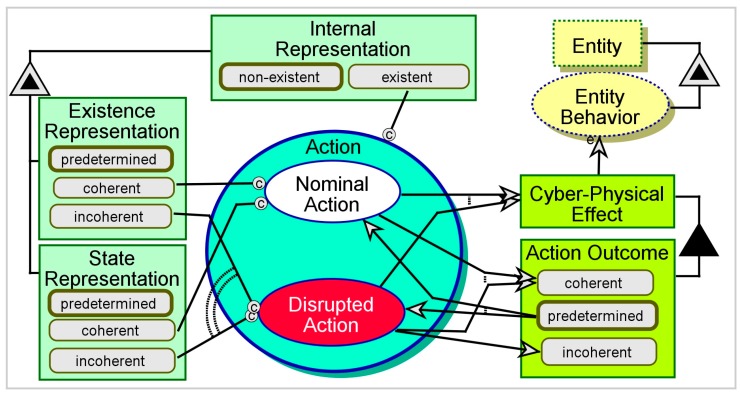
The system’s Nominal Action or Disrupted Action.

Figure 6 illustrates the system’s Action, which results in a **Cyber-Physical Effect**, and includes Nominal Action and Disrupted Action. Disrupted Action may occur instead of or in addition to Nominal Action. Action occurs only if the system has an *existent* **Internal Representation**, since otherwise it has nothing to refer to, even if the external **Entity** is present and engaging the system. Nominal Action occurs if both the **Existence Representation** and the **State Representation** are *coherent*. Otherwise, Disrupted Action occurs. Obviously, Nominal Action ends in a *coherent* **Action Outcome**. Disrupted Action can result in either a *coherent* or an *incoherent* **Action Outcome**. The **Cyber-Physical Effect** triggers and affects the external Entity Behavior. A *coherent* **Cyber-Physical Effect** results in nominal, coherent Entity Behavior. An *incoherent* **Cyber-Physical Effect** may be met with coherent Entity Behavior, depending on the **Entity**’s robustness, friendliness, and intelligence, which are not specified in this diagram. Regardless of the designer’s assumptions, the system must analyze the results and assess the interaction’s success.

### 3.6. Interaction Analysis

The third aspect of CPG-awareness and third phase of CPGAME is Interaction Analysis. In the first two phases—Entity Acquisition and Representation Management—the system had to acquire information about the **Entity**. When the CPS exhibits the functionality of interaction with an external entity, it must analyze this interaction and determine whether it has occurred the way it was intended, and whether its results match the intended or expected results. This analysis can be conducted in real-time or immediately thereafter, depending on the system, interaction, and result criticality.

If the internal representation is incoherent, i.e., any of **CPG Type A1**, **CPG Type A2**, **CPG Type B1**, or **CPG Type B2** is *active*, undesired interaction pattern or undesired results can occur. Failure to obtain the intended or expected interaction is defined as **CPG Type C1**, while failure to generate the expected result (regardless of whether the interaction was as planned or not) is define as **CPG Type C2**.

A CPG-aware model has to account for the way the system uses the internal representation that it holds—rather than the actual state of the entity being represented—to interact with the external entity. The model has to cover the impact of incoherent interaction or its incoherent results, while accounting for the possible latency of the occurrence or detection of the impact of interaction. Finally, the model has to cover techniques to identify and mitigate **CPG Type C1** and **CPG Type C2**.

**Figure 7:**
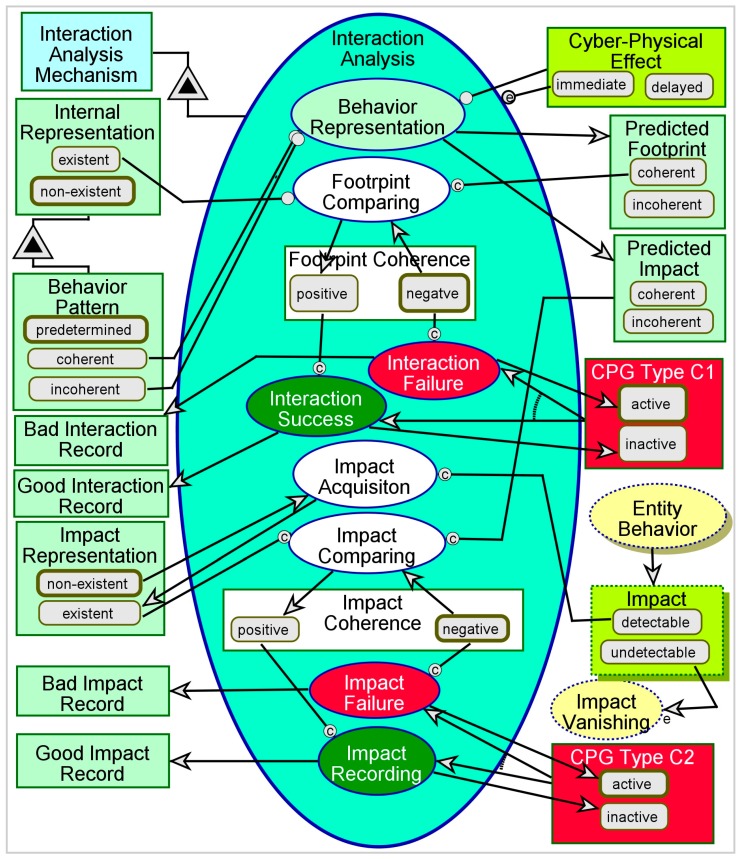
The Interaction Analysis process.

Acquiring the interaction’s course and outcome is similar to the acquisition of Entity behavior and the **Entity**’s **State**, as we can see in Figure 7. As shown, this procedure is triggered by the **Cyber-Physical Effect**, which represents the impact of the system’s Action on the **Entity**. An *immediate* **Cyber-Physical Effect** triggers Interaction Analysis, while a *delayed* effect means waiting until some external event changes it to *immediate*. There are two main parts in this procedure—first, determining whether the interaction itself is as intended, as inferred from internally simulating the Behavior Representation; second, determining whether the result of the interaction is as intended, as indicated by the **Impact** of the **Entity** on the **Environment**. The outcome of the first part is a determination of **CPG Type C1**—failure to conduct the interaction as expected. The outcome of the second part is a determination of **CPG Type C2**—failure to obtain the desired result or intended impact. The reason for incoherent interaction is most likely incoherent representation of the entity, since the action taken by the system as part of interaction with the entity is based on the internal representation.

Coherent representation may still result in incoherent interaction. This may imply that the interaction model is invalid—i.e., the system expects a result which is not feasible. For instance, imagine a central computer network control system that shuts down endpoint terminals to save energy. The control system orders some endpoint terminal to shut itself down when it is active—and known to be active by the central control—but the terminal does not shut itself down since a user working on that terminal manually disables that action on the terminal’s side. In such a case, even though the representation is coherent, the interaction is not as expected, because the control system did not account for the possibility that a user may interfere with the interaction. Correct representation of the user as a separate and independent entity may help resolve this problem.

Interaction coherence and impact coherence are not completely dependent. Incoherent interaction may still result in a coherent impact if the interaction is within the tolerance boundaries for the impact to occur, or if the entity is intelligent or robust enough to compensate for the incoherent interaction. In addition, the desired result may be obtained by coincidence. For this reason, we do not set coherence values after interaction success or failure determination, as well as after impact success or failure determination. Rather, we issue a message or indication—**Good Interaction Record**, **Bad Interaction Record**, **Good Impact Record**, and **Bad Impact Record**—which can be used to drive a different process to investigate or find a root cause for any anomaly.

### 3.7. Model Completeness

Appropriate and sufficient coverage of the system-of-interest by the model that represents it is a critical objective, even regardless of CPD considerations. The pattern shown in the above sections covers the necessary treatment for each entity in the environment of the system. Furthermore, it is the necessary treatment for each entity in each subsystem’s environment, which includes the ambient environment of the top-level system, including the peer-subsystems, in addition to the external environment. Hence, we derive the criterion for complete CPG-awareness as Proposition 1.

**Proposition** **1.** Each entity E(i) must be handled in a CPG-aware manner by any other entity E(j), i,j = 1,..,N.

A CPG-aware manner of handling an external entity, as required in Proposition 1, depends on compliance with the CPGAME pattern. It is necessary to have means (mechanisms and operations) for: (1) entity acquisition, (2) representation management, (3) representation-based action, reaction, or interaction, and (4) interaction analysis. In addition, (5) an internal representation must be defined, and any attribute, state, and behavior must be represented as part of it. The CPG-awareness level for each pair of entities (i,j) is equal to the average of compliance indicators compliance(i,j,k) for the above criteria, $k=1,..,K_{Criteria};\;K_{Criteria}=$ 5.

Some pairs of entities might be infeasible or irrelevant, while some might be of higher importance and criticality. Each pair in which the first entity is a strictly physical object, with no sentience or intelligence, is irrelevant. We may also prefer to neglect second-order CPG-awareness, i.e., awareness of an internal mechanism—which is also an entity—that was created to provide CPG-awareness in another entity. We may wish to neglect pairs of informatical components that are members of the same higher-level informatical entity, and exclude pairs of environmental entities, unless we have to understand how they interact in order to close a control loop or reason about it. We multiply the compliance result for each pair of entities by the pair’s importance factor w(i,j).

Finally, we divide the CPG-awareness score by the maximum possible score, to obtain the relative CPGA index in percentage, as defined in Equation (1):

(1) $$CPGA=\frac{\sum_{i=1}^{N_{Entities}}\sum_{j=1}^{N_{Entities}}\left(w\left(i,j\right)\cdot \sum_{k=1}^{K_{Criteria}}compliance\left(i,j,k\right)\right)}{\sum_{i=1}^{N_{Entities}}\sum_{j=1}^{N_{Entities}}w\left(i,j\right)\cdot N_{Criteria}}\cdot 100\%$$

We seek to ensure maximum coverage of potential CPGs, especially those that do not stand out immediately. Theoretically, a model may achieve a CPGA score of 100% if all the relevant entity pairs in the model are fully compliant with the CPGAME pattern. However, this does not necessarily mean that the model covers all possible CPGs in the problem domain. This challenge corresponds to the area of uncertainty called “unknown unknowns”—uncertain factors, which are not identified or explicitly pointed out in advance, and could have significant impacts if they materialize [55]. The understanding that there are unknown unknowns in any system is a key insight. This mandates securing placeholders for unidentified factors at each level in the system model that account for any potential residual uncertainty. The OPM model should therefore include at least one *unknown* state for each entity, and at least one **Unknown Entity**. These will encourage search for additional CPGs that result from lack of identification or modeling of entities, preventing the CPGA from reaching its theoretical maximum or completeness. In turn, lack of model completeness due to incomplete knowledge motivates continuous search of knowledge gaps.

## 4. Applying CPGAME to the TMI2 Accident

The Three Mile Island nuclear reactor partial meltdown accident of 28 March 1979 (TMI2) is the severest accident in the history of US commercial nuclear power plants. The accident occurred at the nuclear facility located on Three-Mile Island, Susquehanna River, near Middletown, Pennsylvania. The TMI reactor consists of two nuclear pressurized water reactor units, which are active to this day. We have decided to analyze the TMI2 accident due to the fact that, apart from a simple mechanical failure in a relief valve, the primary reason for the results is in fact CPG: the actual state of the physical valve was not understood in real-time by the reactor control team. Due to that mismatch, the operators made erroneous decisions and took wrong actions, which severely exacerbated the already dire situation, resulting in partial meltdown [26]. Today, nuclear power plants must ensure compliance with various power-level control, safety, operability, and availability regulations, in order to preserve economic viability and public acceptance [56]. However, Defense-in-Depth, which is defined by the US Nuclear Regulatory Commission as “an approach to designing and operating nuclear facilities that prevents and mitigates accidents that release radiation or hazardous materials” [57], has been criticized for its deficiency in observing accident factors [13,26]. The course of events that led to a partial nuclear meltdown is described in Table 3, based on the description of events in US Nuclear Regulatory Commission website [14].

### 4.1. Modeling the TMI Reactor System

We have built an OPM model of the TMI reactor system and TMI2 accident. The model was gradually evolved through three model versions:

V1: The first, *naïve* model version, is the reactor’s nominal model, in which the reactor functions appropriately with no issues.
V2: The second, *fault-aware* model, describes the failure that led to the accident, but cannot cover or predict the crisis scenario due to indifference to the CPG.
V3: The third, *CPG-aware* model version captures the CPG of various types and wrong decisions that were made during the emergency and contributed to it.

We compare the three versions and show how CPGAME upgrades the ability to capture and simulate the CPG that caused TMI2. All the versions of the model are available on-line at https://1drv.ms/f/s!AsN2SH2tvCOWjmAwBGtBcUqlzcAF (usage is permitted under the Creative Commons CC BY-NC 4 license terms) for further experimenting and analyzing.

### 4.2. The Naïve Model

**Figure 8:**
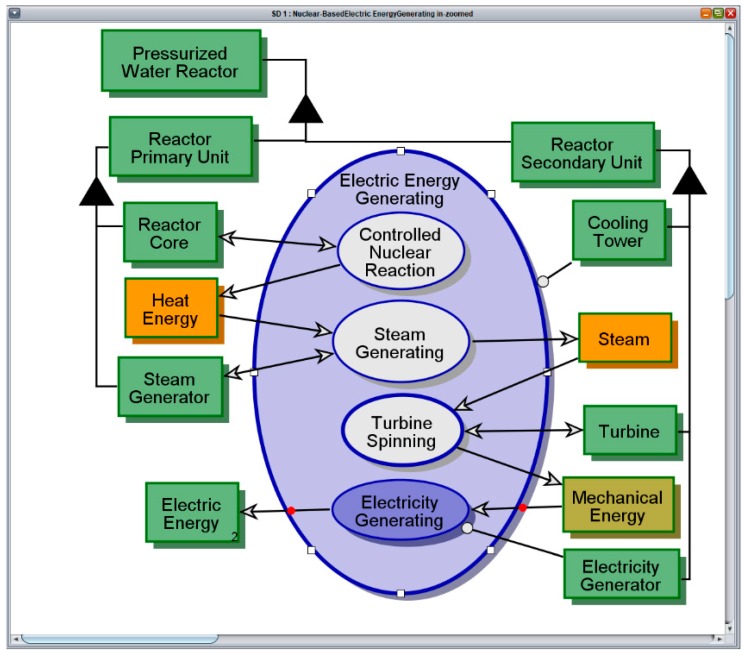
Electric Energy Generating using a pressurized water reactor—nominal operation.

The graphical view of the TMI Reactor naïve model is shown in Figure 8. The current version of the model covers the nominal, failure-free operation of a **Pressurized Water Reactor**, and its main function, Electric Energy Generating. Each iteration of this cyclic process consists of the following four stages: (1) Controlled Nuclear Reaction transforms **Nuclear Fuel** to **Heat Energy**; (2) Steam Generating transforms the **Heat Energy** to **Steam**; (3) Turbine Spinning transforms **Steam** to **Mechanical Energy**; and finally; (4) Electricity Generating transforms **Mechanical Energy** to **Electric Energy**. The model is captured in simulation mode, while the fourth and final stage in the cycle is executing. The naïve model covers the Nominal Action^ (the caret indicates references to things in the generic pattern model), which is the default option of the Action^ of the **System^** as shown in the pattern model in Figure 6. Currently the model does not account for any sensory activity.

### 4.3. The Fault-Aware Model

Having constructed the nominal reactor model, we gradually extend it to cover the possible faults and failures that led to the meltdown accident. This stage can help visualize and simulate first-order failure modes, but it does not yet make any distinction between the physical failure and its identification. It is still assumed that a physical fault is directly and immediately identified by the system. To some extent, this is still a naïve approach, as it ignores the perception gap, but the model is still more informed than the nominal model.

**Figure 9:**
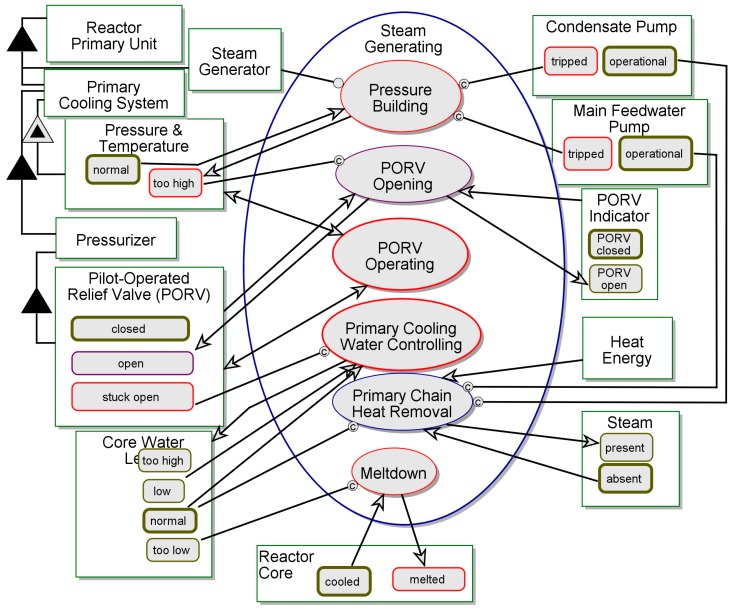
Steam Generating—Fault-Aware Model.

We relax the assumption that resources, instruments, inputs, or outputs in the model are always in a nominal state. A fault-aware in-zoomed view of Steam Generating from Figure 8 is shown in Figure 9. The disrupted objects, states, and processes are painted in red. Most of the fault-aware Steam Generating now specifies possible failure modes and anomalies. This view intentionally captures states and activities that are intuitively not supposed to be in a model: one does not expect a meltdown event, for instance, in a nuclear reactor’s functional model. The concern raised by the addition of such a disturbing possibility to the model is secondary to the insight generated by understanding the impacts of such adverse events while analyzing the model. The *stuck-open* **Pilot-Operated Relief Valve (PORV)** is another example. It may imply component reliability issues, which designers often prefer to conceal. Highlighting such an issue is exactly how the model enhances overall system reliability and provides important information on critical failure modes. Note that the *possibility* of a failure or mismatch is what interests and intrigues us, more than its *probability*.

### 4.4. The CPG-Aware Model

The CPG was a critical factor in the TMI nuclear reactor operators’ decision to decrease water supply to the reactor core. The operators were not aware of the fact that after the PORV stuck, water was still pouring out of the reactor core, causing coolant starvation and reactor overheating. Thinking instead that there is excess water due to the wrong PORV indication, in order to avoid dangerous core vibrations, they shut down whatever emergency water that was still flowing to cool down the core, sealing its fate.

**Figure 10:**
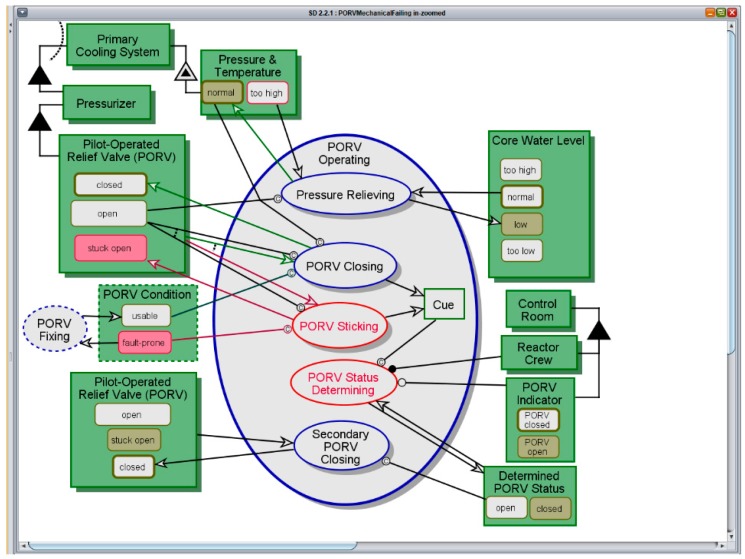
PORV Operating—CPG-Aware Model.

Figure 10 shows a screenshot of running OPCAT simulation of the CPG-aware model, while the PORV Operating process is executing. This process is a more robust replacement for PORV Mechanical Failing in the fault-aware model in Figure 9, taking place after PORV Opening. During the process, the **PORV** becomes *stuck-open*, instead of *closed*, due to its *fault-prone* **PORV Condition**. At the same time, the **PORV Indicator** reads *open*, but the **Determined PORV Status** is set to *closed* by mistake. The **Determined PORV Status** constitutes an **Internal Representation** of the corresponding attribute of the **PORV**, which is an **External Entity** for the control system. In this case there is a mismatch between the actual state of the physical entity and the perceived state of the representation. We have classified this situation as **CPG Type B1^**. If the **Determined PORV Status** were set to *open*, regardless of the state of **PORV Indicator**, it would have led to Secondary PORV Closing, and the control team could have saved the day. This could have happened if the water that was flowing out through the PORV were monitored, rather than the mechanical state indicator. Hence, this is also a case of **CPG Type A1^** with respect to the escaping water. This examples also highlights the importance of (a) capturing the human operator’s understanding of the situation, and not only of the output that is provided by the system to the operator; and (b) specifying viable, robust, and reliable detection solutions that would provide direct rather than indirect indications.

The CPG in the TMI2 accident was in fact double. After the PORV indication was misread, a wrong conclusion was made about the amount of water in the reactor, relying on the assumption that since the PORV is closed then the amount of water is probably sufficient. No sensor for direct measuring of the water level in the core was used. The water level was wrongly assessed based on indirect indication.

**Figure 11:**
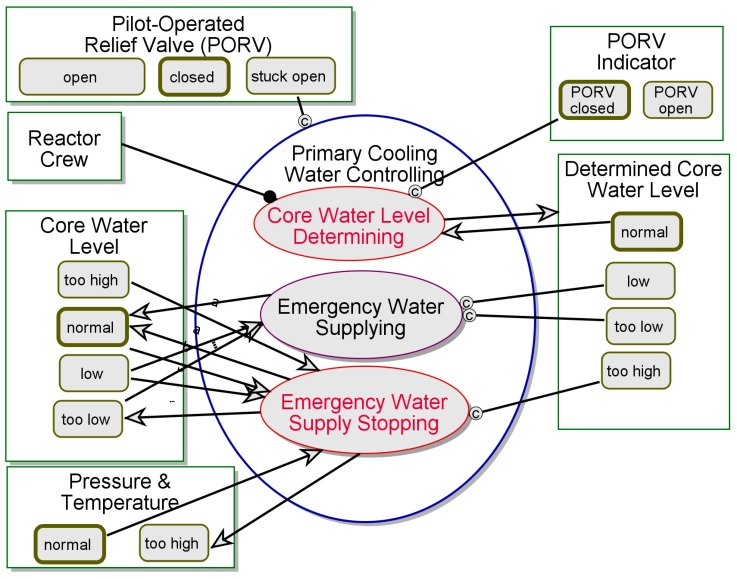
Primary Cooling Water Controlling—CPG-Aware Model.

In Figure 11 we zoom into Primary Cooling Water Controlling, a more robust replacement of Primary Cooling Water Depleting, which appears in Figure 9 after PORV Mechanical Failing, and follows PORV Operating. This process takes place only if the **PORV** is *stuck-open*, hence this is a Disrupted Action. First, the **Determined PORV Status** reads *closed*, so Core Water Level Determining occurs and sets **Determined Core Water Level** to *too high*, while it is in fact *normal* or even *low*. This is the second **CPG Type B1^**, in which the wrong estimation of the water level was reached. If the **Determined PORV Status** had read *open*, the Emergency Water Supplying process could have taken place, the actual **Core Water Level** could have been balanced, and safety could have been restored. However, setting Determined Core Water Level to *too high* caused the opposite—Emergency Water Supply Stopping occurs, causing the **Core Water Level** to be *too low*, and the **Pressure & Temperature** to be *too high*. This is also both a case of **CPG Type B2^**, due to the failure to simulate and predict the behavior of the system once water supply is stopped, and a case of **CPG Type C1^**, due to the failure to perform the intended interaction with the reactor and the water.

**Figure 12:**
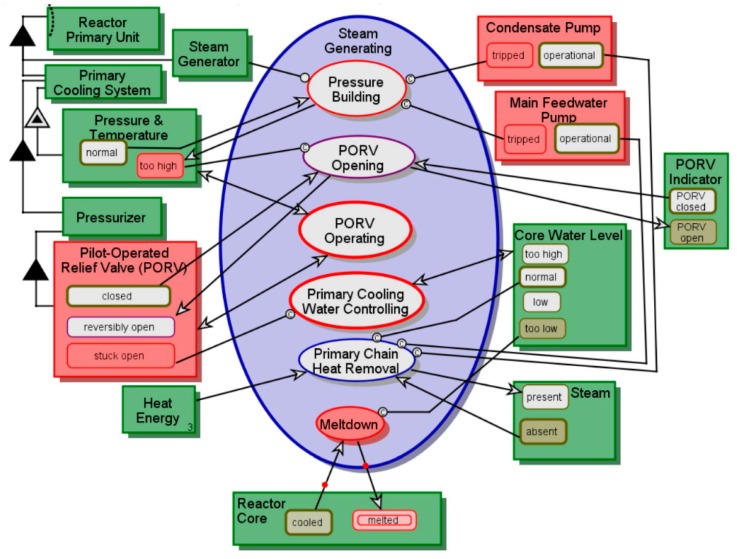
Steam Generating—Meltdown—CPG-Aware Model.

The result is shown in Figure 12, which captures the model’s running simulation while executing the Meltdown process, which only takes place if **Core Water Level** is *too low*. This situation is never supposed occur, but we can simulate and mitigate it by improving the design thanks to the CPG-aware modeling and simulation. The *melted* **Reactor Core** state is a manifestation of **CPG Type C2^**—failure to obtain the intended impact on the physical system.

The textual OPL specification of the model, which is equivalent to the graphical view, is available in the shared model on-line folder.

### 4.5. Enhanced Model Evaluation

We evaluate the contribution of the CPG-aware OPM model relative to the nominal model through several perspectives. First, we compare the number of statements in each model in order to determine the rate of improvement in the informativity of the model. A comparison of the V1 and V3 is summarized in Table 4. V2 is an interim version, and is therefore omitted from the comparison. The total number of statements in V3 (186) more than tripled itself compared to V1 (60). Especially noticeable is the growth in the number of behavioral statements (from 25 to 97), due to the CPG-associated procedure and conditionality specification in the context of the stuck-open PORV and the adverse results. Two specific statement kinds with notable growths are State-set Definition (from 1 to 13) and Condition Link (from 0 to 18). The growth in states and conditions marks: (i) the focus shift from general structure and process specification in V1 to situational and conditional modeling in V3; and (ii) the evolvability of the OPM model to cover these aspects. In addition, the model revision started as a focused elaboration on a specific failure mode (stuck-open PORV), but necessitated many additional and complementary modifications and extensions to cover the problem and course of events that we were trying to capture. This simple comparison clearly shows that a CPG-aware model is significantly more informative than its nominal counterparts, if only due to the idea that disruption-informed modeling is self-expanding, as multiple implications, considerations, and complementary aspects arise once the model is constructed this way.

The TMI2 CPG-aware model directly covers 5 of 6 CPG types: A1, B1, B2, C1, and C2. CPG Type A2 is covered implicitly or indirectly by this example, since the reactor system’s failure to detect the escaping water keeps the CPG Type A2 active by default. Table 5 summarizes the six CPG types and how they were demonstrated in the TMI2 example.

The CPGA index, defined in Section 3.7, provides an assessment of the level of CPG-awareness as reflected by the model. One can see that the original, naïve model (V1) had a *CPGA* of 0, since it had no reference to CPG or CPG-awareness. In the CPG-aware model (V3), we can calculate *CPGA*, but we first need to determine which entity pairs are applicable for *CPGA* scoring. The model contains N = 52 objects and processes in V3 (excluding simulation-supporting things). The possible number of entity pairs is therefore N∙(N-1), which is 2652. However, we focus on two primary CPG-handling agents: **Control Room** and **Reactor Crew**. We assume that all the control and decision processes in the system are carried out by the **Reactor Crew** in the **Control Room**. Hence the number of applicable entity pairs is only 2∙(N-1), which is 102. We have included CPG mechanisms for three entity pairs: **Pilot-Operated Relief Valve (PORV)**, represented in the **Control Room**’s KB as **PORV Indicator**, **PORV Indicator**, represented in the **Reactor Crew** perception as **Determined PORV Status**, and **Core Water Level**, represented in the **Reactor Crew** perception as **Determined Core Water Level**. Note that **PORV Indicator** is both an internal representation in the **Control Room** and an external entity for the **Reactor Crew**.

The compliance of each pair of entities with the CPG criteria is summarized in Table 6. All three pairs have an internal representation, acquisition, and action. There is no explicit process for generating the **PORV Indicator**. Furthermore, we only have two interaction analysis processes. Therefore, the $CPGA=\left(\frac{3}{5}+\frac{4}{5}+\frac{4}{5}\right)/102=2.16\%$. This score is relatively low for this model, but it only covers the failure modes that led to the TMI2 accident, and hence cannot serve as an estimate for the scope of CPG-aware handling in the system.

## 5. Discussion and Conclusions

CPGAME establishes a well-defined distinction between an entity—a user or actor, another subsystem, an asset, or a resource—and its representation. Since the entity in the model is already a representation of either the real entity or the informatical representation, creating the additional abstraction layer has been challenging. The CPGAME approach facilitates representation-based interaction between the system and the external entity. The pattern that CPGAME provides enables modeling and subsequent model-based handling of anomalies, such as lost, untracked, or misperceived physical objects. The more the cyber-physical environment is unreliable and inconsistent, the more critical it is to apply the CPGAME approach. Although CPGAME adds a layer of complexity to the system, accounting for CPG increases the accuracy, fidelity, reliability, safety, and security of the system, and consequently the overall performance and robustness levels of systems in general and those of safety-critical systems in particular.

Integrating CPG-aware design elements into existing system models is also challenging. As we show in the TMI2 case study, OPM facilitates extending nominal models to make them CPG-aware. Ignoring CPG-related problems, or failure to address them during design time, gives them a ‘green light’ to show up unexpectedly during system operation, often in the worst time possible and with potentially dire, even detrimental consequences. The TMI2 case is a prime example of both CPG in complex CPSs and the significant impact that CPGAME can have on predictability and mitigation of risks and other adverse effects. It also shows that CPG may appear not only in modern “cyber-rich” systems, but also in legacy systems. Hence, these systems should receive special attention from owners and regulators in an attempt to identify and mitigate CPG related risks before they materialize. CPGAME can be applied for reverse engineering of legacy systems, discovering potential CPGs, and auditing the actual system.

One limitation of the current CPGAME pattern is its relatively deterministic nature. It assumes that as part of the system’s CPG-control mechanism, the characteristics of the external entity—its attributes, states, and behaviors, are either not represented at all or fully represented. Future research should remove this limitation, possibly by incorporating into the model fuzzy logic representation.

Future research involves the study of additional cases, in which the CPG was or may have been a primary factor for systems’ dysfunctional or disrupted behavior, in an attempt to derive characteristics of CPG-prone systems and predict CPG in systems based on these characteristics. One such case, the Malaysia Airlines 370 disappearance, is obviously related to CPG in the air traffic control systems that should have followed the flight when it disappeared. While a thorough audit of this case would require access to confidential or classified control systems and records, the theoretical explanation of this case in light of the CPG and using CPGAME would be a major contribution to the investigation of the case by air safety authorities and agencies, and would benefit stakeholders in similar conditions around the globe.

In addition, we intend to demonstrate CPGAME on a variety of system analysis cases, review the designs of existing systems, and determine whether they are exposed to CPGs and whether that exposure could cause an impact on system behavior or its outcomes. In such a case, we would propose ways and mechanisms to mitigate the potential adverse impacts of the CPG.

Another obviously valuable application of CPGAME would be validation, verification, testing, and auditing of systems, especially CPG-prone ones. As part of model-based testing, future research should also investigate efficient utilization of CPGAME for verification of system behavior, resilience, and robustness when CPGs materialize.

## Figures and Tables

**Table 1:** Levels of Systemic Conception of Reality.

Conception Level	KB Awareness	IB Perception
Kind	The system knows the definition of the entity kind.	The system is informed of the existence of a specific instance of the known entity kind.
Feature	The system knows that there is a possibility of existence of a significant feature—a part, an attribute, or an operation of the entity.	The system is informed of the existence of a specific item of the known entity feature.
State-Space	There is a possible set of states or range of values that the entity’s attribute can assume.	The system is informed of the specific state or value of a part, attribute, or operation of a specific entity.

**Table 2:** Classes and Types of CPG.

Class	Type
Class A: failure to detect or identify a detectable entity	Type A1: detection mechanism problem or lack of a pattern that resembles the entity
	Type A2: acquisition mechanism problem or inability to perform entity pattern-matching
Class B: failure to generate a coherent representation of an acquired entity	Type B1: problem with representation of the acquired entity’s state
	Type B2: problem with representation of the acquired entity’s behavior
Class C: failure to generate a coherent interaction with an acquired entity	Type C1: problem with generating the intended interaction
	Type C2: problem with generating the intended result of an interaction

**Table 3:** Three-Mile Island 2 accident course of events [14].

#	Event	Effect
1.	A failure in secondary section of the plant prevented the main feedwater pumps from providing coolant to steam generators.	The steam generators could not help cool the reactor core.
2.	The turbine-generator and reactor automatically shut down.	Pressure in primary, nuclear unit, began to increase.
3.	Pilot-operated relief valve (PORV) opened.	Pressure dropped
4.	PORV closed.	PORV stuck halfway through (“stuck-open”).
5.	Instruments in control room indicated that PORV was closed.	Operators were unaware of the cooling water’s pouring out through stuck-open valve.
6.	Control instruments did not indicate how much water was covering the core.	Operators assumed that as long as pressurizer water level was high, the core was properly covered with water.
7.	Alarm rang due to coolant loss, core exposure and overheating.	Operators do not identify loss-of-coolant accident.
8.	Water escaped through faulty PORV and reduced pressure too much	Core got to risk of dangerous vibrations.
9.	Operators reduced emergency coolant input to primary.	Core is starved of coolant and overheats.
10.	Without sufficient cooling water, the nuclear fuel overheated	Nuclear fuel pellet cladding ruptured and they start melting.
11.	Someone noticed another indicator of stuck-open PORV, closed emergency valve	Cooling water stopped pouring out of reactor; reactor gradually stabilized.

**Table 4:** TMI2 Comparative Analysis of Model Versions.

Measure	Nominal Version (V1)	CPG-Aware Version (V3)	Growth Rate
Total statements	60	186	+126 (210%)
Structural statements	35	89	+54 (154%)
State-set Definition	1	13	+12 (1200%)
Behavioral statements	25	97	+72 (288%)
Condition Link	0	18	+18

**Table 5:** TMI2 model coverage of CPG cases.

CPG Type	Demonstrated	How/Why
A1 (No Detection)	Yes	Water escaping through PORV not detected.
A2 (No Acquisition)	Indirectly	Water escaping through PORV not acquired.
B1 (State Representation)	Yes	Determined PORV status vs. actual PORV status Determined water level vs. actual PORV status
B2 (Behavior Representation)	Yes	Predicted water and core behavior due to emergency water supply stopping
C1 (Interaction)	Yes	Water level depleting, rather than steadying, after emergency water stopping
C2 (Impcat)	Yes	Meltdown, rather than core stabilizing

**Table 6:** TMI2 model compliance with CPGA criteria.

Systemic Agent	External Entity	Internal Representation	Acquisition	Representation	Representation-Based Action	Interaction Analysis	Total Compliance
**Control Room**	**Pilot-Operated Relief Valve**	**PORV Indicator**	assumed to be found in KB	naive	Secondary PORV Closing	missing	3/5
**Reactor Crew**	**PORV Indicator**	**Determined PORV Status**	assumed to be found in KB	PORV Status Determining	Secondary PORV Closing	Primary Cooling Water Controlling	4/5
**Reactor Crew**	**Core Water Level**	**Determined Core Water Level**	acquired indirectly (CPG Type A1+A2)	PORV Water Level Determining	Emergency Water Supplying	Primary Chain Heat Removal; Meltdown	4/5

## Abbreviations

CPD	Cyber-Physical Duality
CPG	Cyber-Physical Gap
CPGAME	Cyber-Physical Gap-Aware Modeling and Engineering
CPS	Cyber-Physical System(s)
MBSE	Model-Based Systems Engineering
OPD	Object-Process Diagram
OPL	Object-Process Language
OPM	Object-Process Methodology
SysML	Systems Modeling Language
TMI	Three-Mile Island
UML	Unified Modeling Language
